# Image File Management to Support International Telepathology

**DOI:** 10.1155/2014/913423

**Published:** 2014-11-19

**Authors:** Liron Pantanowitz, Jeffrey McHugh, William Cable, Chengquan Zhao, Anil V. Parwani

**Affiliations:** ^1^Department of Pathology, University of Pittsburgh Medical Center, USA; ^2^Department of Information Services, University of Pittsburgh Medical Center, USA

## Background

Telepathology practice across international borders has become increasingly popular. Two years after the University of Pittsburgh Medical Center (UPMC) launched a telepathology consultation service with KingMed Diagnostics Laboratory in Guangzhou, China, latency issues occurred when viewing whole slide images (WSI). Our aim was to explore various image file management solutions to improve the viewing experience of digital consult cases.

## Methods

WSI files generated by KingMed when scanning glass slides for consultation initially resided on a Hamamatsu server, using the NDP.serve database management system. These remote files were securely accessed using a custom portal via the Internet. To try solving ongoing latency issues, WSI files were instead transferred from China to the UPMC data center using an open source product (Fast Data Transfer, developed by CERN). This was a command line utility placed into a batch process. A faster high speed file transfer software solution (Aspera) was subsequently employed. This commercial software allowed immediate file transfers to occur without a user initiating the transfer.

## Results

Viewing digital consult cases from Pittsburgh in the USA with WSI files residing on a server in China negatively impacted viewing of images. Image display deteriorated to about 2 minutes/case. Transferring files with the open source product provided transfer speeds of 2-3 MB/second but suffered from intermittent dropped connections. Employing the commercial software permitted more reliable transmission of digital files with 75–100 MB/second transfer speeds.

## Conclusion

Successful global telepathology requires dedicated image management. Transfer of files to local servers delayed the process by up to 24 hours but greatly improved the overall turn-around time of digital consultations. This was partly negated by employing high speed file transfer software. Transfer of digital files helped overcome network latency issues experienced with China and enhanced the viewing experience for end-user digital consultants.

